# *Genetics Selection Evolution* reviewer acknowledgement 2014

**DOI:** 10.1186/s12711-015-0147-z

**Published:** 2015-09-24

**Authors:** Didier Boichard, Jack Dekkers, Helene Hayes, Julius van der Werf

**Affiliations:** INRA, UMR 1313 Génétique Animale et Biologie Intégrative, 78350 Jouy-en-Josas, France; Department of Animal Science and Center for Integrated Animal Genomics, Iowa State University, Ames, IA USA; School of Environmental and Rural Science, University of New England, Armidale, Australia

## Abstract

The *Genetics Selection Evolution* editorial team would sincerely like to thank all of our reviewers who contributed to peer review for the journal in 2014.

**Syuiti Abe**

Japan

**Mark Abney**

United States of America

**Hervé Acloque**

France

**Samuel E. Aggrey**

United States of America

**Andreia Amaral**

Portugal

**Peter Amer**

New Zealand

**Leif Andersson**

Sweden

**Jesus Arango**

United States of America

**Benoit Auvray**

New Zealand

**William Barendse**

Australia

**C. Simon Bawden**

Australia

**Bertrand Bed'Hom**

France

**Gary Bennett**

United States of America

**Jörn Bennewitz**

Germany

**Camillo Berenos**

United Kingdom

**Donagh Berry**

Ireland

**Jean-Pierre Bidanel**

France

**Piter Bijma**

Netherlands

**Marco Bink**

Netherlands

**Stephen Bishop**^^^

United Kingdom

**Solomon Antwi Boison**

Austria

**Agnes Bonnet**

France

**Lanie Bourgeois**

United States of America

**Gudrun Brockmann**

Germany

**Fanny Calenge**

France

**Mario Calus**

Netherlands

**Rodolfo Juan Carlos Cantet**

Argentina

**Aurélien Capitan**

France

**Maria Carabaño**

Spain

**Roberto Carvalheiro**

Brazil

**Eduardo Casas**

United States of America

**Alessio Cecchinato**

Italy

**Carole Charlier**

Belgium

**Claude Chevalet**

France

**Ole F. Christensen**

Denmark

**Christian Edel**

Germany

**Elena Ciani**

Italy

**Samuel Adam Clark**

Australia

**Noelle Cockett**

United States of America

**John Cole**

United States of America

**Wouter Coppieters**

Belgium

**Pascal Croiseau**

France

**Ron Crump**

United Kingdom

**Ino Curik**

Croatia

**Yang Da**

United States of America

**Hans D. Daetwyler**

Australia

**Binyam Sime Dagnachew**

Norway

**Ingrid David**

France

**Dirk-Jan de Koning**

Sweden

**Gustavo de los Campos**

United States of America

**Jared Decker**

United States of America

**Julie Demars**

France

**Olivier Demeure**

France

**Leo Dempfle**

Germany

**Xiangdong Ding**

China

**Joerg Dodenhoff**

Germany

**Tom Druet**

Belgium

**Susana Dunner**

Spain

**Mathilde Dupont-Nivet**

France

**Klaus Ehrhardt**

Germany

**Jean Michel Elsen**

France

**Jeffrey Endelman**

United States of America

**Malena Erbe**

Germany

**Catherine Ernst**

United States of America

**Stéphane Fabre**

France

**Bin Fan**

China

**Pierre Faux**

Belgium

**Majbritt Felleki**

Sweden

**Jesús Fernández**

Spain

**Rohan Fernando**

United States of America

**Laurence Flori**

France

**Sylvain Foissac**

United States of America

**Luca Fontanesi**

Italy

**Alexis Fostier**

France

**Brad Freking**

United States of America

**Luis Alberto García Cortés**

Spain

**Jose-Luis García-Marín**

Spain

**Dorian Garrick**

United States of America

**Giustino Gaspa**

Italy

**Mathieu Gautier**

France

**Helene Gilbert**

France

**Linus Girdland Flink**

United Kingdom

**Luis Gómez-Raya**

Spain

**Oscar González-Recio**

Spain

**Gregor Gorjanc**

United Kingdom

**Martien Groenen**

Netherlands

**Bernt Guldbrandtsen**

Denmark

**Satoshi Hamaguchi**

Japan

**Bevin Harris**

New Zealand

**Ben Hayes**

Australia

**Susanne Hermesch**

Australia

**Juan Manuel Herrero-Medrano**

Netherlands

**William Hill**

United Kingdom

**Jisca Huisman**

United Kingdom

**Noelia Ibañez**

Spain

**Eveline Ibeagha-Awemu**

Canada

**Ikhide Imumorin**

United States of America

**Jean-Luc Jannink**

United States of America

**Luc Janss**

Denmark

**Steven Janssens**

Belgium

**John William Keele**

United States of America

**Kathryn Kemper**

Australia

**Brian Kinghorn**

Australia

**Brian Kirkpatrick**

United States of America

**Byung-Whi Kong**

United States of America

**Loeske Kruuk**

Australia

**Larry Kuehn**

United States of America

**Robert Lacy**

United States of America

**Laurence Lamothe**

France

**Catherine Larzul**

France

**Jan Lassen**

United States of America

**Tosso Leeb**

Switzerland

**Andres Legarra**

France

**Christina Lehermeier**

Germany

**Grégoire Leroy**

France

**Debby Lipschutz-Powell**

United Kingdom

**Zengting Liu**

Germany

**Daniela Lourenco**

United States of America

**Tu Luan**

Norway

**Peipei Ma**

China

**Daniel Maizon**

Argentina

**Mahlako Makgahlela**

South Africa

**Christian Maltecca**

United States of America

**Zissis Mamuris**

Greece

**Esa Mantysaari**

Finland

**Christel Marie-Etancelin**

France

**Raluca Mateescu**

United States of America

**John McEwan**

New Zealand

**Ivica Medugorac**

Germany

**Gábor Mészáros**

Austria

**Theo Meuwissen**

Norway

**Ignacy Misztal**

United States of America

**Philippe Monget**

France

**Raphael Mrode**

United Kingdom

**Han Mulder**

Netherlands

**Sebastian Munilla**

Argentina

**Riccardo Negrini**

Italy

**Pablo Orozco-terWengel**

United Kingdom

**Mikhail Ozerov**

Finland

**Hubert Pausch**

Germany

**Francisco Penagaricano**

United States of America

**Paulino Pérez**

Mexico

**Miguel Pérez-Enciso**

Spain

**Florence Phocas**

France

**Jeffrey Plowman**

New Zealand

**Ricardo Pong-Wong**

United Kingdom

**Laercio R Porto-Neto**

Australia

**Jennie Pryce**

Australia

**Herman Raadsma**

Australia

**Sebastian Ramos-Onsins**

Spain

**Wendy Mercedes Rauw**

Spain

**Denis Réale**

Canada

**Kent Reed**

United States of America

**Norbert Reinsch**

Germany

**Carlo Renieri**

Italy

**Marcio Resende**

United States of America

**Anne Ricard**

France

**Silvia Teresa Rodriguez Ramilo**

Spain

**Andrés Rogberg-Muñoz**

Argentina

**Gary Rohrer**

United States of America

**Lars Rönnegård**

Sweden

**Sophie Rothammer**

Germany

**Suzanne Rowe**

New Zealand

**Goutam Sahana**

Denmark

**Juan Pablo Sanchez**

Spain

**Magali San Cristobal**

France

**Anna Wensley Santure**

United Kingdom

**Honoo Satake**

Japan

**Larry Schaeffer**

Canada

**Karl Schmid**

Germany

**Dierck Segelke**

Germany

**Anna K. Sonesson**

Norway

**Tad Sonstegard**

United States of America

**Daniel Sorensen**

Denmark

**Juan Steibel**

United States of America

**Ismo Stranden**

Finland

**Guosheng Su**

Denmark

**Bolormaa Sunduimijid**

Australia

**Richard Tait**

United States of America

**Miika Jani Juhani Tapio**

Finland

**Frank Technow**

United States of America

**Robert Tempelman**

United States of America

**Jens Tetens**

Germany

**Elizabeth Thompson**

United States of America

**Bruce Tier**

Australia

**Miguel Angel Toro**

Spain

**Thierry Tribout**

France

**Daniel Vaiman**

France

**Julius van der Werf**

Australia

**Marc Vandeputte**

France

**Paul Vanraden**

United States of America

**Luis Varona**

Spain

**Roel Veerkamp**

Netherlands

**Johanna Vilkki**

Finland

**Beatriz Villanueva**

Spain

**Renaud Vitalis**

France

**Zulma Vitezica**

France

**Chunkao Wang**

United States of America

**Ji-Ping Wang**

United States of America

**Joel Ira Weller**

Israel

**Robin Wellmann**

Germany

**Pam Wiener**

United Kingdom

**Yvonne Wientjes**

Netherlands

**George Wiggans**

United States of America

**Klaus Wimmers**

Germany

**Anna Wolc**

Poland

**Kui Zhang**

United States of America

**Qin Zhang**

China

